# Rapid and Synchronous Breeding of Cytoplasmic Male Sterile and Maintainer Line Through Mitochondrial DNA Rearrangement Using Doubled Haploid Inducer in *Brassica napus*

**DOI:** 10.3389/fpls.2022.871006

**Published:** 2022-04-26

**Authors:** Wei Zhang, Haoran Shi, Ying Zhou, Xingyu Liang, Xuan Luo, Chaowen Xiao, Yun Li, Peizhou Xu, Jisheng Wang, Wanzhuo Gong, Qiong Zou, Lanrong Tao, Zeming Kang, Rong Tang, Zhuang Li, Jin Yang, Shaohong Fu

**Affiliations:** ^1^Chengdu Academy of Agricultural and Forestry Sciences, Chengdu, China; ^2^Chengdu Research Branch, National Rapeseed Genetic Improvement Center, Chengdu, China; ^3^Agricultural College, Sichuan Agricultural University, Chengdu, China; ^4^Maize Research Institute, Sichuan Agricultural University, Chengdu, China; ^5^Key Laboratory of Bio-Resource and Eco-Environment of Ministry of Education, College of Life Sciences, Sichuan University, Chengdu, China; ^6^Rice Research Institute, Sichuan Agricultural University, Chengdu, China

**Keywords:** double haploid inducer, rapid breeding, cytoplasmic male sterile lines (CMS), maintainer lines, mitochondrial DNA

## Abstract

When homozygously fertile plants were induced using doubled haploid (DH) induction lines Y3380 and Y3560, the morphology of the induced F_1_ generation was basically consistent with the female parent, but the fertility was separated, showing characteristics similar to cytoplasmic male sterile (CMS) and maintainer lines. In this study, the morphology, fertility, ploidy, and cytoplasm genotype of the induced progeny were identified, and the results showed that the sterile progeny was *polima* cytoplasm sterile (*pol* CMS) and the fertile progeny was *nap* cytoplasm. The molecular marker and test-cross experimental results showed that the fertile progeny did not carry the restorer gene of *pol* CMS and the genetic distance between the female parent and the offspring was 0.002. This suggested that those inductions which produced sterile and fertile progeny were coordinated to CMS and maintainer lines. Through the co-linearity analysis of the mitochondrial DNA (mtDNA), it was found that the rearrangement of mtDNA by DH induction was the key factor that caused the transformation of fertility (*nap*) into sterility (*pol*). Also, when heterozygous females were induced with DH induction lines, the induction F_2_ generation also showed the segregation of fertile and sterile lines, and the genetic distance between sterile and fertile lines was approximately 0.075. Therefore, the induction line can induce different types of female parents, and the breeding of the sterile line and the maintainer line can be achieved through the rapid synchronization of sister crosses and self-crosses. The induction of DH inducer in *B. napus* can provide a new model for the innovation of germplasm resources and open up a new way for its application.

## Introduction

*Brassica napus* belongs to the *Cruciferae* (Chalhoub et al., 2014) and evolved as an allo-tetraploid crop from *Brassica rapa* (AA, 2*n* = 20) and *Brassica oleracea* (CC, 2*n* = 18) through interspecific hybridization and natural doubling (NU, 1935; Palmer et al., 1983; Bancroft et al., 2011; Sun et al., 2017). Since *B. napus* has two sets of heterologous chromosome sets (A/C; Olsson, 1960; Leflon et al., 2006; Allender and King, 2010; Chalhoub et al., 2014; Hu et al., 2021), the cycle of selfing to obtain homozygous lines is long. Although anther or microspore culture techniques can obtain homozygous line plants (Keller and Armstrong, 1978; Lichter, 1982; Kott et al., 1998; Xu et al., 2007; Shariatpanahi and Ahmadi, 2016) and have the potential to develop improved varieties (Custers, 2003; Ferrie and Caswell, 2011), they are influenced by some conditions such as culture temperature (Custers et al., 1994), plant development stage (Takahata et al., 1991), and genotype of donor plants (Takahata et al., 1996; Barro and Martín, 1999; Li and Devaux, 2001). *B. napus* is an early crop to use CMS lines for heterosis utilization (Fu et al., 1990). In 1973, Prof. Fu Tingdong from Huazhong Agricultural University in China discovered *polima* cytoplasm male sterile line (*pol* CMS) (Fu, 1981), which opened the prelude of heterosis utilization of *B. napus* (Witt et al., 1991). Although the traditional cytoplasmic three lines technology (maintainer line, CMS line, restorer line) has been applied to rapeseed production on a large scale (Fu et al., 1989, 1990; Li et al., 1997), the breeding and improvement of CMS had a long and difficult cycle, requiring 6–12 generations. Generally, the new CMS lines are bred by backcrossing stable or largely stable maintainer lines in pairs for multiple generations with the original CMS lines. The application of biotechnology, such as gene editing, has enabled the rapid creation of male-sterile lines and maintainers, but the technology is difficult at the research stage because of the stability and transgenic safety issues (Du et al., 2020).

Since the discovery of haploids in *Datura stramonium* in the early 1920s (Blakeslee et al., 1922), the methods to obtain pure lines by haploid induction lines and distant hybridization have been widely used in maize (Pollacsek, 1992; Zhao et al., 2013), rice (Niizeki and Oono, 1968), wheat (Laurie and Bennett, 1986; Laurie, 1989), *Arabidopsis* (Ravi et al., 2014), and other plants (Burk et al., 1979; Forster et al., 2007; Chen et al., 2011b). The induction efficiency of haploid induction lines represented by maize “*Stock 6*” was about 2% (Coe, 1959). Now the efficiency of haploid induction has been increased to more than 10% by improvement (Eder and Chalyk, 2002; Geiger and Gordillo, 2009; Rotarenco et al., 2010). Therefore, *in vivo* haploid induction and artificial chromosome doubling can obtain homozygous lines more quickly and easily (Chase, 1952; Palmer et al., 1996; Jacquier et al., 2020). In recent years, Fu et al. (2018) artificially synthesized two allo-octoploid (AAAACCCC, 2*n* = 8X ≈ 76) rapes of doubled haploid induction lines, Y3560 and Y3380, to provide a new way for obtaining stable pure lines (maintainer line or restorer line) in rapeseed. Y3560, Y3380, and their parents P3-2 have changes in the methylation patterns of pollen (Yin et al., 2021) and have abnormal meiosis (Yin et al., 2020) to produce aneuploid gametes, which exhibited strong induction ability (Luo et al., 2021). When the DH induction lines were used as the male parent to pollinate different genotypes of *B. napus*, the induction effect was found to be influenced by maternal karyogene and cytoplasmic genotype (Zhang et al., 2021). Therefore, it suggests differences in the induction mechanism of DH induction lines in *B. napus* by haploid induction in maize and *Arabidopsis*. Meanwhile, DH inducer can be used to quickly obtain interspecific hybrid offspring and broaden genetic resources (Zhou et al., 2022), and it can realize multiple gene homoeologs editing of *B. napus* and *B. oleracea* using the DH induction line Y3380-mediated editing system, which brings hope to rapeseed and cruciferous vegetable breeding that are difficult to be gene-edited through genetic transformation (Li et al., 2021). In the process of induction, it was found that when the DH induction line Y3380 and Y3560 induced homozygously fertile parents, the morphology of the induced F_1_ generation plants was almost similar to that of the female parent, but sterile and fertile segregation occurred, showing similar characteristics of CMS and maintainer lines. During the induction process, it was found that the morphology of the F_1_ generation plants induced by the DH induction lines Y3380 and Y3560 when inducing homozygous fertile parents was basically similar to that of the female parents (Zhang et al., 2021). However, the F_1_ generation showed segregation of sterile and fertile plants in terms of fertility, while exhibiting similar characteristics of CMS and maintainer lines. Based on these phenomena, we performed morphological and cytological observations combined with molecular markers to identify the fertility and cytoplasm genotypes between parents and offspring before and after induction. We further performed mitochondrial DNA co-linearity analysis with genome resequencing and mitochondrial genome assembly. These data will provide a theoretical basis for rapid and simultaneous breeding of CMS lines and maintainer lines, and provide a new insight for accelerating and enriching the use of hybrid advantage in *B. napus*.

## Materials and Methods

### Plants Materials and Cultivation Conditions

Information on the main plant materials for this study is presented in Table 1, in which the control male parent 20–2,386 was a normal tetraploid *pol* restorer line (F_22_). The female parent 0933A was *pol* CMS (BC_15_). Z1732 was their hybrid F_1_. The rape DH induction lines Y3560 and Y3380 were bred by Fu Shaohong of Chengdu Academy of Agriculture and Forestry Sciences through artificial synthesis in 2011 and were identified as allo-octoploid rapes by cytology and flow cytometry (AAAACCCC, 2*n* = 8x≈ 76; Fu et al., 2018). The 0933B is the 0933A maintainer line and is a normal tetraploid *pol* maintainer line (F_20_). ZY21-1 and ZY21A-1 are the induced progeny with 0933B as the female parent and Y3560 as the male parent. The female parent L0933A is normal *ogu* CMS (BC_10_) and the maintainer line is 0933B, which is the same as *pol* 0933A maintainer line. ZY26A-1 is a hybrid hexaploid progeny with L0933A as the female parent and the induction line Y3380 as the male parent (Table 1). To avoid mixing during planting, all planting material were planted in pots at a young stage and each plant was marked. Plants were subsequently transplanted to the field after the determination of plant ploidy by flow cytometry. All plants were hand-pollinated at flowering and seeds were obtained by single threshing. The plant material in this experiment was grown under the same conditions in October 2019 in an experimental field in Wenjiang, Chengdu, China (E103.83, N30.70).

**TABLE 1:** Test material information.

Material category	Material name	Material use	Fertility	ploidy	Material source
Rape DH induction lines	Y3560, Y3380	Paternal parent	Fertile	Octoploid	Provided by Chengdu Academy of Agriculture and Forestry Sciences
*Pol* recovery line	20-2386		Fertile	Tetraploid	
*Nap* maintainer line	0933B	Female parent (maintainer line corresponding to sterile line)	Fertile		
*pol* CMS	0933A		Sterility		
*ogu* CMS	L0933A		Sterility		
Induced F1	ZY21-1		Fertile		0933B × Y3560
	ZY21A-1		Sterility		
Hybrid F1	ZY26A-1		Sterility	Hexaploid	L0933A × Y3380
	Z1732		Fertile	Tetraploid	0933A × 20-2836
Induced heterozygous female parent F2	4233, 4232, 3987, 3852		Fertile		Induced heterozygous *pol* female parent
	4233A, 4233A, 3987A, 3852A		Sterility		
	3925, 3926, 3928		Fertile		Induced heterozygous *pol* nucleus sterile female parent
	3925A, 3926A, 3928A		Sterility		
	3821, 3823, 3824, 3911		Fertile		Induced heterozygous *ogu* nucleus sterile female parent
	3821A, 3823A, 3824A, 3911A		Sterility		

### Field Identification and Sampling of Plant Morphology and Fertility

Based on the fertility characteristics, fertile or sterile was identified as a marker trait by manual observation in the field. The morphology of *pol* CMS plants was not significantly different from that of the normal fertile plants, but the anthers were shrunken and there was no pollen or only a small amount of pollen at low temperature, while *ogu* types of sterile lines did not produce pollen. According to the results of fertility identification, the individual plants with the same morphology as the female parent were sampled and stored at −80°C for future use.

### DNA Extraction and PCR Amplification

All parents and offspring associated with this study were identified by molecular markers. The total DNA was extracted by referring to Doyle’s classical CTAB method. PCR amplification procedure: (1) restorer gene amplification procedure (Zhang et al., 2017): 94°C, 5 min, 94°C, 30 s, Tm, 30 s, 72°C, 45 s (Goto Step2, 35 cycles), 72°C, 8 min, 10°C for holding. (2) Cytoplasmic primer amplification program 94°C, 4 min, 94°C, 30 s, Tm, 30 s, 72°C, 1 min (Goto Step2, 24 cycles), 72°C, 8 min, 4°C keep warm. The setting of Tm was according to different primers (Heng et al., 2015). Among the cytoplasmic identification primers (Table 1), MSS4, MSS8, and MSS14 identified *nap*, *pol*, and *ogu* cytoplasm, respectively (Heng et al., 2015).

### Chromosome Number Identification in Somatic Cells

Young flower buds of F_1_-generation (ZY21-1, ZY21A-1, and ZY26A-1) plants were treated with 8-hydroxyquinoline solution, placed in the dark for 3 h, and then fixed by Carnot fixative (ethanol: glacial acetic acid, 3:1) for 24 h and stored in 70% ethanol. Alcohol was removed with distilled before observation followed by immersion in 1 mol/L hydrochloric acid in a water bath at 60°C for 8–10 min. Carbol magenta staining was used to determine chromosome numbers under the microscope. Cytogenetic observations were made according to the process detailed in Fu et al. (2018).

### Pollen Viability Identification

Fresh flower bud of different fertile progeny (ZY21-1, ZY21A-1) of the F_1_ generation at the blooming stage was collected separately. The anthers were placed on slides, crushed with forceps, and then 1–2 drops of magenta acetate solution were placed on coverslips, gently pressed, and observed under the microscope. When pollen grains are dark red, they are viable and light red indicates partial loss of viability; colorless, hollow, or deformed are dead and sterile pollen grains. Cytogenetic observations were made according to the process detailed in Yin et al. (2020).

### Anther Freezing Section

Three flower buds of different lengths from different fertility plants (ZY21-1, ZY21A-1) in the induced F_1_ were selected separately. The anthers were separated from the flower buds and dehydrated. The dehydrated anthers were placed in an embedding box containing 1/3 of the embedding solution and frozen at −20°C. After complete solidification, 1/3 of the embedding solution was added and frozen at −20°C for complete solidification and then prepared for use. The slides were stained with eosin staining solution for about 15 s. The slides were washed with water to remove excess staining solution, dried, and placed under a microscope for observation and storage.

### Flow Cytometry Detection

Fresh young leaves of the tested plants (all induced offspring) were quantitatively taken with a 5-mm diameter punch and placed in a pre-chilled culture dish. Then 0.5 ml of pre-chilled cell lysis buffer was added and the leaves were quickly chopped and filtered through a 300-mesh filter in a 2-ml EP tube. A total of 1.5 ml PI (propidium canonical) staining solution (50 μg/mL PI, 50 μg/mL RNase) was added and stored for 30 min in a place protected from light. The samples were analyzed using a flow cytometer (Accuri^®^ C6 Plus, BD) and analyzed using the accompanying Accuri C6plus software. The specific operation procedure is described in Yin et al. (2020).

### Whole-Genome Resequencing

Sampling was performed by manually identifying the fertility of the plants (a total of 32 materials are involved). DNA was extracted from the collected plant leaves, and the DNA samples of acceptable quality were sent to Biomarker Technologies Co., Ltd. for next-generation sequencing at a depth of 10X. Sequencing data were used to calculate Nei genetic distance for SNP data and to perform inter-sample cluster analysis.

### Assembly and Annotation of the Mitochondrial Genome

**FIGURE 1:**
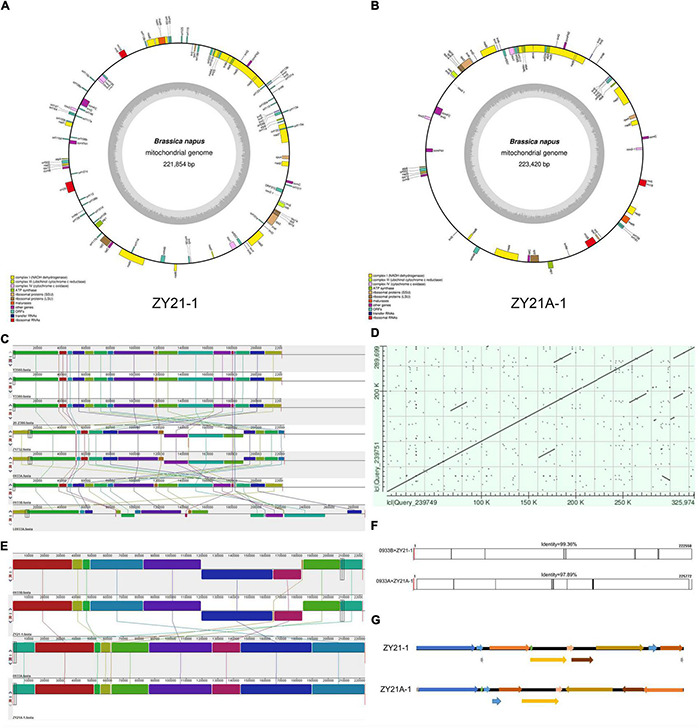
Results of mitochondrial genome co-linearity analysis for different samples. **(A)** Annotated map of the mitochondrial genome of induced F_1_ generation fertile progeny ZY21-1. **(B)** Annotated map of the mitochondrial genome of induced F_1_ generation sterile progeny ZY21A-1. **(C)** Schematic diagram of mitochondrial genome co-linearity between parental and hybrid F_1_ generation before and after induction, from top to bottom, Y3560, Y3380, 20-2386, Z1732, 0933A, 0933B, L0933A. **(D)** Comparison of the mitochondrial genome between hybrid hexaploid ZY26A-1 and its female parent L0933A. **(E)** Schematic diagram of mitochondrial genomic covariance between induced F_1_ generation and 0933A and 0933B, from top to bottom, 0933B, ZY21-1, 0933A, ZY21A-1. **(F)** Comparison of mitochondrial sequences between 0933B and ZY21-1; comparison of mitochondrial sequences between 0933A and ZY21-1. **(G)** Comparison of mitochondrial genome arrangement of different fertile single plants in the induced F_1_ generation ZY21-1and ZY21A-1.

The analysis of the mitochondrial genome mainly involved a total of 10 materials (Y3380, Y3560, 0933A, 0933B, L0933A, ZY21-1, ZY21A-1, ZY26A-1, 20-2386, and Z1732). Based on the raw data obtained from whole-genome resequencing, sequences were analyzed and extracted according to the mitochondrial genome characteristics (Albertsen et al., 2013; Zhou et al., 2013), and finally, the assembly and annotation (Supplementary Figure 3 and Figures 1A,B) of the mitochondrial genome was completed by Wuhan Yiersan Biotechnology Co., Ltd. The primers (Supplementary Table 1) were designed to amplify and sequence at both ends of the linked overlapping cluster contig to verify the splice region and obtain the complete mtDNA (Supplementary Figures 2B–D). The covariance between mitochondrial genomes was analyzed using NCBI Blast,^1^ co-linearity analysis software Mauve. DNAman was also used to detect the identity between mitochondrial genomes. Among them, the induced lines Y3560, Y3380, 20-2386, and Z1732 unified the cut positions to compare the sequence alignment among mitochondrial genomes more clearly (Figures 1C–F).

### Functional Annotation and Enrichment

The mutation loci were screened in whole-genome re-sequencing and ZS11 was used as a reference to annotate and enrich the genes where the mutation loci are located. Subsequently, GO and KEGG annotation information^2^ were integrated using Perl language. The KEGG annotation results were also enriched through the KEGG online website.^3^

## Results

### Flower Morphology and Cytoplasm Genotype Identification

**FIGURE 2:**
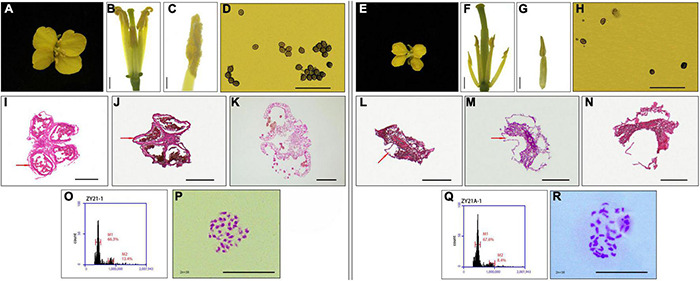
Fertility and ploidy identification of different fertility progeny (ZY21-1 and ZY21A-1) in the induced F_1_ generation. **(A)** Flower morphology of induced F_1_-generation fertile progeny ZY21-1. **(B,C)** Anther morphology of induced F_1_-generation fertile progeny (ZY21-1), **(B)** bar = 2mm, **(C)** bar = 1mm. **(D)** Pollen activity assay results of induced F_1_-generation fertile progeny (ZY21-1). **(E)** Flower morphology of induced F_1_ generation sterile progeny ZY21A-1. **(F,G)** Anther morphology of induced F_1_ generation sterile progeny (ZY21A-1), Bar = 2mm. **(H)** Pollen activity assay results of induced F_1_ generation sterile progeny (ZY21A-1). **(I–K)** Induced F_1_ fertile progeny (ZY21-1) with different flower bud lengths (0.2–0.25cm, 0.25–0.3cm, > 0.3cm) of anther slices, in which the red-brown arrow points to the tapetum layer. Bar = 100μm. **(L–N)** Induced F_1_ sterile progeny (ZY21A-1) with different flower bud lengths (0.25–0.3cm, 0.3–0.4cm, > 0.4cm) of anther slices. The position indicated by the red-brown arrow indicates that the tapetum layer has degenerated. Bar = 100μm. **(O)** Flow cytometry results of induced F_1_ generation fertile progeny ZY21-1. **(P)** Number of the chromosome of induced F_1_ generation fertile progeny ZY21-1, Bar = 10μm. **(Q)** Flow cytometry results of induced F_1_ generation sterile progeny ZY21A-1. **(R)** Number of the chromosome of induced F_1_ generation sterile progeny ZY21A-1, Bar = 10μm.

**FIGURE 3:**
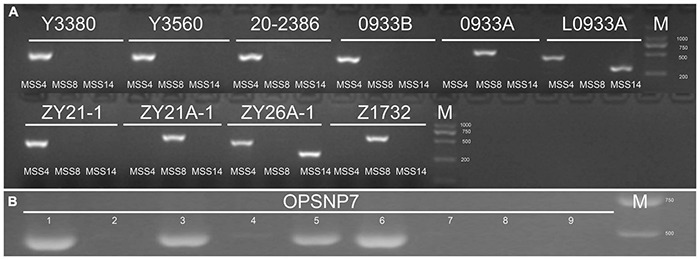
Identification results of cytoplasmic genotype and pol CMS recovery gene *Rfp1*. **(A)** Results of cytoplasmic identification with primers MSS-4 (*nap*), MSS-8 (*pol*) and MSS-14 (*ogu*). **(B)** Results of recovery gene identification, 1–9, respectively: 20-2386; 0933A control; Y3380-1, Y3380-2, Y3560-1, Y3560-2, 0933B, ZY21-1, ZY21A-1, and OPSNP7 are primers for the specific amplification of *pol* CMS restorer gene *Rfp1*.

It was found that the induced F_1_ generation plants produced when the induction lines induced the homozygous female parent were consistent with the female parent in morphology and ploidy, but the segregation for fertility had occurred (Zhang et al., 2021). Therefore, the induced F_1_ generation ZY21-1 (fertile) and ZY21A-1 (sterile) with different fertility were identified for the homozygous maintainer line (0933B, fertile) as the female parent and the induction line Y3560 (fertile) as the male parent. The floral organs of the fertile progeny ZY21-1, including sepals, petals, stamens, and nectaries were normally developed (Figures 2A–C). They had high pollen fertility (Figure 2D) and the tapetum was able to develop normally to produce spores (Figures 2I–K). In contrast, the sterile progeny ZY21A-1 had reduced floral organ size, slightly ruffled petals, reduced anther length, shrunken stigma (Figures 2E–G), and only a small amount of pollen (Figure 2H), which might be due to the degeneration of the tapetum resulting in abnormal microspore development (Figures 2L–N); consequently no pollen or only a small amount of pollen was produced (Figures 2F–H). Since the DH induction line Y3560 is an allo-octoploid *B. napus*, pollination to the tetraploid fertile plant 0933B could produce ploidy hybridization, resulting in chromosome increase or elimination, leading to the development of sterility. Further, the ploidy and chromosome observation of the sterile and fertile progeny after induction showed that the ploidy of the induced F_1_ generation fertile progeny ZY21-1 (Figure 2O) and sterile progeny ZY21A-1 (Figure 2Q) was consistent, and they were both tetraploid (400.0–500.0D thousand lines) with 38 chromosomes (Figures 2P,R). The results indicated that the appearance of sterile plants has not resulted from chromosome gain or loss after ploidy hybridization. To further clarify whether the cytoplasm genotypes of the induced F_1_ generation were changed, the cytoplasm genotypes of the parents and the offspring before and after induction were identified by cytoplasm identification primers (Heng et al., 2015). The results showed that the induction lines Y3560 and Y3380 were *nap* cytoplasm and the female parental 0933B was *nap* cytoplasm, 0933A was *pol* cytoplasm (0933B was the maintainer line of the high generation self-cross of 0933A), and L0933A was *ogu* cytoplasm (Figure 3A). Further, the hybrid F_1_ generation Z1732 (fertile) and ZY26A-1 (sterile) were identified (Figure 3A), and the results showed that the cytoplasm of the hybrid progeny (Z1732, ZY26A-1) was the same as the cytoplasm of the female parent (0933A, L0933A). The fertile progeny ZY21-1 of the induced F_1_ generation was *nap* cytoplasm, and the sterile progeny ZY21A-1 was *pol* CMS (Figure 3A), indicating that the CMS progeny ZY21A-1 that appeared in the induced F_1_ generation was consistent with the speculation of the flower morphology observation.

In conclusion, the induction effect of the DH inducer was different from the crosses of parents with the same ploidy level. When the homozygous female parent 0933B with *nap* cytoplasm was induced, the flower morphology and cytoplasmic genotype of the sterile progeny were identified as *pol* CMS, that is, cytoplasmic changes occurred after induction.

### Nei Genetic Distances Revealed a Relationship in the Nuclear Background of the Maternal Parent and Offspring Before and After Induction

Due to the induction effect of the induction line, if 0933B converts *nap* cytoplasm into *pol* CMS, would the corresponding karyogene also change? Therefore, SNP loci (2296756 ∼ 3102779) of different samples from whole-genome resequencing data at 10x depth were examined and the Nei genetic distances (Table 2) were calculated by SNP loci data to assess the nuclear background between the maternal parent and offspring before and after induction. The results showed that the genetic distance between 0933B (maintainer lines) and 0933A (*pol* CMS) was only 0.002 (Table 2), while 0933B and 0933A were the maintainer (BC_15_) and CMS lines with the same nuclear background. However, 0933B and L0933A were genetically distant (0.026) (Table 2). The number of the back cross was less than that of 0933B and 0933A (BC_10_); therefore it was reliable to detect the genetic relationship between samples by Nei genetic distance. Subsequently, it was found that when 0933B was the female parent, the genetic distances between the induced F_1_ generation ZY21-1 and ZY21A-1 and 0933B were 0.002 (Table 2), indicating that the nuclear backgrounds between the induced F_1_ generation and the female parent were almost identical. However, the hybrid hexaploid progeny ZY26A-1 (Supplementary Figure 1) with L0933A as the female parent and the induction line Y3380 as the male parent had a genetic distance of 0.377 (Table 2) from the female parent.

**TABLE 2:** SNP locus estimates Nei genetic distance between parents and progeny.

	0933B	0933A	L0933A	ZY21-1	ZY21A-1	ZY26A-1
0933B						
0933A	0.002					
L0933A	0.026	0.026				
ZY21-1	0.002	0.002	0.026			
ZY21A-1	0.002	0.002	0.026	0.002		
ZY26A-1	0.371	0.371	0.377	0.371	0.371	

The restorer genes corresponding to the *pol* CMS of the parents and offspring before and after induction were examined (Figure 3B and Supplementary Figure 2A), and it was found that the induction lines Y3560 and Y3380 carried the *pol* CMS restorer gene *Rfp1* (Zhang et al., 2017), but there was segregation between different individual plants, probably due to genetic instability (Fu et al., 2018). The induced F_1_ generation fertile progeny ZY21-1 and the female parent 0933B did not carry the *pol* CMS restorer gene *Rfp1*. Meanwhile, the hybrid offspring Z1732, which was crossed with *pol* CMS restorer line 20-2386 and *pol* CMS 0933A, carried the restorer gene. These data indicate that the induction of the induction lines occurs and can cause changes in plant fertility and cytoplasm without affecting the nuclear background of the induced progeny, which will lay the foundation for the rapid and simultaneous acquisition of the maintainer lines and the corresponding sterile lines.

### Co-linearity Analysis of Parental and Offspring Mitochondrial DNA

The Nei genetic distance indicated that the nuclear background between the female parent and offspring before and after induction was almost identical (Table 2), but the molecular marker identification results indicated that ZY21A-1 cytoplasm in the induced F_1_ generation was changed (Figure 3A). To further know whether the corresponding mtDNA controlling fertility is also changed and whether the production of sterile plants is caused by changes in the mitochondrial genome, we assembled and analyzed mtDNAs from a total of 10 samples before and after induction using whole-genome resequencing data (Figures 1A,B and Supplementary Figure 3). First, the mtDNAs of three female parents (0933B, 0933A, L0933A; Supplementary Figures 2B–D, 3A–C), the three male parents (DH induction lines Y3380, Y3560, *pol* restorer line 20-2386) (Supplementary Figures 3E–G), and one hybrid F_1_ generation (Z1732) (Supplementary Figure 3H) in this study were analyzed for co-linearity (Figure 1C), and it was found that the mtDNAs of the three male parents and the 0933B (*nap*) were almost identical, while the mtDNAs of the different female parents 0933B (*nap*), 0933A (*pol*,) and L0933A (*ogu*) differed significantly in the arrangement due to different cytoplasm genotypes (Figure 1A). However, the similarity in mitochondrial genome sequences between 0933A (*pol*) and 0933B (*nap*) was higher (Figure 1C), and the detection results were consistent with the molecular identification results and could be mutual verification.

Secondly, the hybrid F_1_ results showed that the mitochondrial genome of the F_1_ generation Z1732 (Supplementary Figure 3H) of the cross with 0933A (*pol*) (Supplementary Figure 3B) as the female parent and 20-2386 (*nap*) (Supplementary Figure 3G) as the male parent had the same mitochondrial genome sequence arrangement as that of the female parent 0933A (Supplementary Figure 3B). The results indicated that the inheritance of mtDNA in the normal crossing process followed maternal inheritance. In contrast, the mitochondrial genome of the hexaploid progeny ZY26A-1 (*ogu*) (Supplementary Figures 1, 3D), which was generated by crossing the induction line Y3380 (*nap*) (Supplementary Figure 3E) as the male and L0933A (*ogu*) (Supplementary Figure 3C) as the female parent, showed duplication of several fragments and partial sequence inversion compared with the female parent L0933A, which caused rearrangement of the ZY26A-1 mitochondrial genome and a significant increase in the genomic sequence length more than 36,275bp (Figure 1D and Supplementary Figures 3C,D). Subsequently, the mitochondrial genome of the induced F_1_ generation was compared with the same cytoplasmic genome, and it was found that the fertile progeny ZY21-1 had the same mitochondrial genome arrangement as the 0933B (*nap*) (Figure 1E), and the concordance was 99.36% (Figure 1F), while the mitochondrial genome arrangement of the sterile ZY21A-1 was the same as that of 0933A (*pol*) (Figure 1E) and the concordance was 97.89% (Figure 1F). These results showed that the genetic consequences of the crosses and the induction of plants with different ploidy levels and the hybridization of plants with the same ploidy level are different. Crossing and induction between different ploidy might cause changes in the mitochondrial genome, which might be affected by nucleo-cytoplasmic interactions. Together, the mitochondrial genome of the fertile F_1_ generation of ZY21-1 produced by induction of the inducer was the same as that of the female parent 0933B and did not carry the *pol* CMS restorer gene, while the CMS ZY21A-1 was the same as *pol* CMS (0933A). Therefore, since the nuclear background before and after induction is basically the same, it suggests that when the induced line is the male parent, the induced F_1_ generation is the relationship between the corresponding maintainer line (fertile F_1_ progeny) and the CMS line (sterile F_1_ progeny), and this process may be affected by the nuclear–cytoplasmic interaction.

### Verification of Induction and Realization of *Pol* Cytoplasmic Male Sterile and Maintainer Line Synchronous Breeding

**FIGURE 4:**
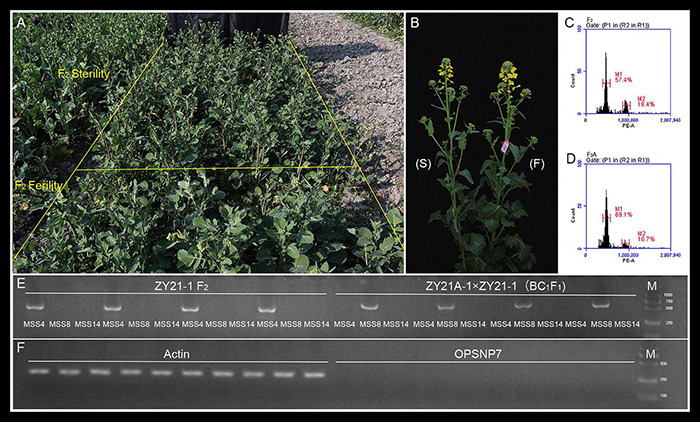
Identification results of different fertility populations (ZY21-1 F_2_ and ZY21A-1 × ZY21-1 (BC_1_F_1_) in the induced F_2_ generation. **(A)** Plant phenotypes of the induced F_2_ generation populations (ZY21-1 F_2_ and ZY21A-1 × ZY21-1 (BC_1_F_1_)) with different fertility populations. **(B)** Plant phenotypes of the different fertile progeny of the induced F_2_ generation population (ZY21-1 F_2_ and ZY21A-1 × ZY21-1 (BC_1_F_1_)), “F,” the fertile progeny ZY21-1 self-crossed F_2_ generation; “S,” the sterile progeny ZY21A-1 × ZY21-1 sister-crossed in the BC_1_F_1_ generation. **(C)** Flow cytometry results of induction of F_2_ generation plant of ZY21-1 self-crossed. **(D)** Flow cytometry results of induction of BC_1_F_1_ generation plant of ZY21A-1 × ZY21-1 sister-crossed. **(E)** Molecular markers result from induced F_2_ and BC_1_F_1_ generation with different fertility population cytoplasm genotypes. **(F)** Molecular markers result of induced F_2_ and BC_1_F_1_ generation *pol* restorer gene, and actin is internal reference gene.

Through the application of various methods for the identification of induced F_1_ generation, it was finally shown that F_1_ generations produced by the inducer were correspondingly related to the CMS lines and the maintainer lines, and the reliability of the identification results by genetic correlation with plant morphology, ploidy, and molecular markers was confirmed. To further verify whether the differences generated by the DH induction lines could be inherited stably in the progeny, we studied self-pollinated progeny of fertile plants from induced F_1_ generation (ZY21-1, F_2_ generation) and the progeny of male–sterile plants obtained by sib-crossing with fertile plants (ZY21A-1 × ZY21A-1, BC_1_F_1_ generation). These progenies had similar growth phenotypes with different fertility (Figures 4A,B), and the ploidy was the same with the tetraploid (Figures 4C,D). The results showed that the cytoplasm genotypes of the induced F_2_ generation were the same as the induced F_1_ generation, and the cytoplasm of the fertile population was *nap* cytoplasm and the sterile population was *pol* cytoplasm (Figures 4E,F), and none of them contained the *pol* CMS restorer gene *Rfp1*. Therefore, the combination of morphology, ploidy, and molecular markers for the identification of the induced F_2_ and BC_1_F_1_ generations showed that the changes produced by the inducer could be inherited stably, and the breeding of the corresponding CMS lines could be achieved simultaneously and rapidly.

### Functional Annotation and Enrichment Analysis of the Genes in Which the Mutant Loci Are Located

To further explore the causes of cytoplasmic type changes, we screened SNP and Indel mutant loci between the parent and the offspring by re-sequencing and a total of 286,552 mutant loci were obtained. Among them, the fertile offspring ZY21-1 produced by induction had 1,064 mutant loci involving 211 genes, the sterile offspring ZY21A-1 had 1,100 mutant loci involving 258 genes, and the hybrid offspring ZY26A-1 had 11,842 mutant loci involving 1,319 genes. The genes where the mutant loci were located were also functionally annotated and enriched. The GO enrichment results showed that the differential loci were mainly enriched in protein binding (Supplementary Figures 4A–C) when the induced lines were used as male parents. However, compared to ZY21-1, ZY21A-1 and ZY26A-1 with significant changes in the mitochondrial genome had more genes related to protein kinase activity and other pathways, and more genes of the same pathway and a wider range of different pathways (Supplementary Figures 4A–C). Also, the KEGG Brite enrichment results showed that the genes corresponding to some mutant loci in the F_1_ generation sterile offspring ZY21A-1 and ZY26A-1 with significant changes in the mitochondrial genome were involved in the mitochondrial biogenesis, while the induced F_1_ generation fertile offspring ZY21-1 without significant changes in the mitochondrial genome was not found to have the same results. In conclusion, the functional annotation and enrichment of the genes at the mutant loci suggest that the induced lines as parents are different from conventional crosses in that they can cause changes in the plants to some extent through protein interactions and in the regulation of the metabolic pathways that eventually feed back to the plants.

### Genetic Distance and Clustering Analysis Among the F_2_ Generation of the Induced Heterozygous Female Parent

**FIGURE 5:**
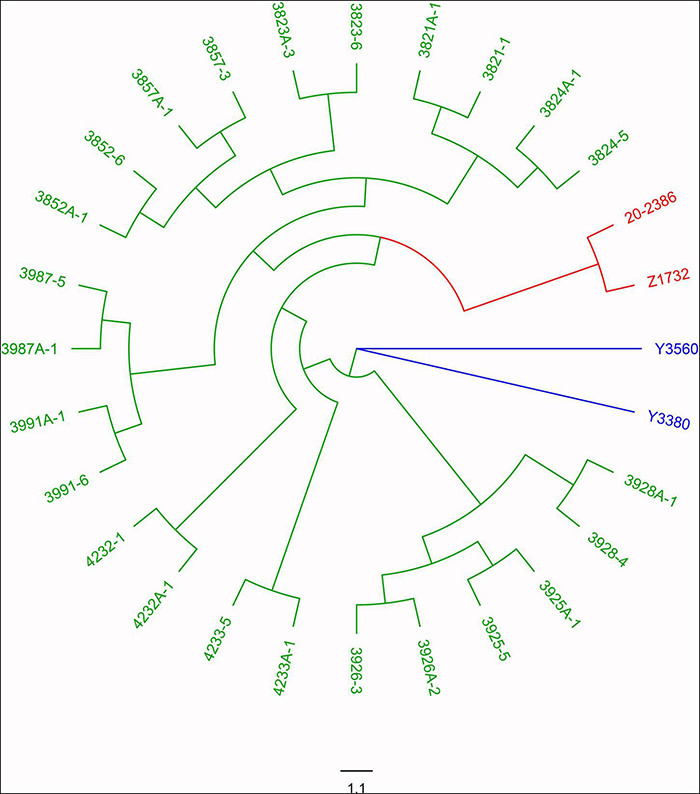
Genetic clustering analysis of F_2_ generations produced by induced heterozygous females. The induction lines Y3560 and Y3380 are in blue, the hybrid control is in red, and the induced F_2_ generation is in green.

The induction of homozygous female parents resulted in the rapid and simultaneous acquisition of both maintainer and sterile populations, but did this phenomenon apply only to the induction of homozygous female parents, can it also occur in heterozygous maternal parents of other cytoplasmic types? To verify whether the induction effect also existed in heterozygous female parent with different cytoplasm types (*pol*, *ogu*), we induced hybrid F_1_ of *pol* and o*gu* cytoplasm using Y3380 and Y3560 as the male parent. The results showed that the induced F_1_ generation was also segregated for fertility, but since the nuclear genes of the female parents were heterozygous, the F_1_ generation itself may also be heterozygous for nuclear genes. To exclude such differences, we self-crossed the induced F_1_ fertile plants from the heterozygous parents to obtain the induced F_2_ generation population. Through the fertility identification of the F_2_ generation population, the sterile plant was 15–34.21% (Supplementary Table 3). Based on these results, the fertile and sterile plants in the induced F_2_-generation population generated by inducing with *pol* and *ogu* cytoplasm types were randomly selected for whole-genome resequencing, and the number of SNP loci between 1857507 and 2273984 from resequencing were identified. The Nei genetic distance was calculated and clustered. The results showed that the genetic distance variation between the hybrid progeny Z1732 and other induced F_2_ generation ranged from 0.487 to 0.334 (Supplementary Table 2), with significant differences in the nuclear background. The genetic distance variation between paired fertile and sterile F_2_ individual plants from the same self-cross induced F_1_ plant ranged from 0.042 to 0.114, with an average of 0.075, and most of the genetic distances were 0.07–0.08 (Supplementary Table 2). The genetic distances between the induced progeny were closer than those between the hybrid progeny, and the nuclear backgrounds of these induced F_2_ generations are thought to be nearly identical between the individual plants of fertility and sterility (Supplementary Table 2). Also, the different fertility plants in the same induced F_2_ generation were clustered due to the same nuclear background, which was consistent with the actual lineage (Figure 5). These results indicated that the induction lines were capable of inducing fertility segregated from both homozygous and heterozygous maternal parents, and the segregated progeny could be rapidly stabilized by self and sister-crosses to eventually form the corresponding maintainer and CMS lines, thus the induction lines had a potential application in achieving simultaneous and rapid breeding of maintainers and CMS lines.

## Discussion

### Rapid Acquisition of Cytoplasmic Male Sterile and Maintainer Lines by Rape Doubled Haploid Inducer

In this study, the parents and offspring were identified before and after induction, and the floral morphology of the sterile progeny ZY21A-1 in the F_1_ generation was highly similar to that of *pol* CMS (Figure 2). Further, molecular marker identification showed that the fertile progeny ZY21-1 in the induced F_1_ generation was *nap* cytoplasm (Figure 3A) and the sterile progeny ZY21A-1 was *pol* cytoplasm (Figure 3A). Also, both of them did not carry the *pol* CMS restorer gene *Rfp1* (Figure 3B). The results of Nei genetic distance showed that the female parent 0933B and the induced F_1_ generation ZY21-1 and ZY21A-1 were all 0.002 (Table 2), and the nuclear background was almost identical. The mitochondrial genomes of the offspring before and after induction were compared with those of the parents. It was found that the mtDNA of the fertile progeny ZY21-1 was 99.36%, which is consistent with ZY21-1 (*nap*). The mtDNA of the sterile progeny ZY21A-1 was 97.89%, consistent with 0933A (*pol)* in the F_1_ generation (Figure 1F). These results are consistent with the identification results with molecular markers.

As a control, the mtDNA of the F_1_ generation of the normal hybrid Z1732 (*pol*) was identical to that of the female parent 0933A (Figure 1C). The genetic distance between the hybrid hexaploid ZY26A-1 and the female parent L0933A was 0.377, with the length of the mitochondrial genome increased by 36,275 bp and with multiple repetitive sequences appearing (Figure 1D and Supplementary Figure 2D). Y3380 and Y3560 were allo-octoploids synthesized by artificial chromosome doubling (Fu et al., 2018), and the size and genotype of the mitochondrial genome are the same as those of tetraploid (Figure 1C and Supplementary Figures 2E,F). This shows that hybridization between different ploidy is different from the same ploidy normal hybridization. The hybridization of plants with the same ploidy is the main hybridization type between karyogenes, and the cytoplasm genes are inherited from the female parent. In addition to karyogenes, cytoplasm genes may change in different ploidy hybridization, and there is an interaction between karyogene and cytoplasm (Zhang et al., 2021). Subsequently, by examining the F_2_ generation population formed from the induced homozygous female parent, it was found that the morphology, ploidy, and cytoplasm genotype in the induced F_2_ generation of different fertility populations were the same as those in the induced F_1_ generation (Figures 4A–F). Simultaneously, the induced F_2_ generation of different fertility plants produced from the induced heterozygous parent also tended to be stable. In summary, the different genotypes of female parents by the induction lines can produce induced progeny with only fertility difference, and the stable populations of maintainers and CMS lines with the same genetic background can be obtained rapidly through self and sister crosses (Figure 5 and Table 2).

### Speculation and Analysis of Induced Cytoplasmic Changes

Numerous studies have shown that allopolyploidization in plants results in rapid genomic changes (Song et al., 1995; Ozkan et al., 2001; Comai et al., 2003; Pires et al., 2004; Soltis et al., 2004; Comai, 2005; Tate, 2006; Gaeta et al., 2007) and leads to chromosomal rearrangements (Pires et al., 2004; Pontes et al., 2004; Chen and Ni, 2006; Leitch and Leitch, 2008), altered DNA methylation (Lewis et al., 2006; Xu et al., 2009; Niederhuth et al., 2016), chromatin remodeling (Leitch and Leitch, 2008), altered gene expression (Chen and Pikaard, 1997; Osborn et al., 2003; Adams and Wendel, 2005; Gaeta et al., 2007), and activation of transposable elements (Kashkush et al., 2002; Ha et al., 2009). The rape DH induction lines by artificial synthesis have similar characteristics (Song et al., 1995; Lewis et al., 2006; Gaeta et al., 2007; Xiong et al., 2011). Also, are the CMS lines produced by the induction lines similar in mechanism to *pol* CMS in *Brassica napus* (Fu, 1981) and wild abortive lines in rice (Yuan, 1986)? In this study, the DH induction lines were used as the male parent and the allo-tetraploid *B. napus* plants were used as the female parent, while causing different genomes to be combined (Szadkowski et al., 2011; Fu et al., 2016). Since male parents have a much higher number of chromosomes than female parents, chromosomes from the male parents are gradually lost during embryonic development (Houben et al., 2011), and there is subsequent doubling of the chromosomes to reorganize the genome dose (Comai et al., 2003). As a result, the endosperm and embryo abortion rates are higher compared with normal crosses, resulting in the formation of a large number of defective seeds (Prigge et al., 2012; Xu et al., 2013; Qiu et al., 2014; Yang et al., 2019), which ultimately retain about 10% of the normal seeds in our experiment (data not shown) (Xu et al., 2013; Melchinger et al., 2017; Luo et al., 2021). The majority of these seeds completely lose the paternal chromosome during development (Houben et al., 2011; Tayeng et al., 2012; Chaudhary et al., 2013), leaving only the maternal chromosome before completing doubling, resulting in homozygous doubled haploid seeds. A small number of these seeds do not completely lose the paternal chromosome (Li et al., 2009; Newaskar et al., 2013; Zhao et al., 2013), resulting in hybrid offspring such as ZY26A-1. However, since the nuclear background between the offspring and the maternal parent is almost identical before and after induction, there is no infiltration or hybridization of large segments of the male parent, but the induced offspring will show partial functional changes and will involve multiple pathways through recombination or interactions. It was shown that transposons are involved in genome recombination in polyploids (Albertin et al., 2006; Ha et al., 2009; Chen, 2010; Zou et al., 2011; Shen et al., 2014), and so it is more reasonable to explain the gain (Gao et al., 2016) or loss of function of the induced progeny by jumping of polyploidy-induced transposons (Fu et al., 2016; Vicient and Casacuberta, 2017). It is further speculated that during induction, recombination genomes induce activation or jumping of nuclear genomic transposons while activating related genes in the maternal cytoplasm genome (Shen et al., 2015; Fu et al., 2016). Also compared to *ogu* cytoplasm, *nap* and *pol* cytoplasm undergo less rearrangement and recombination in the sequence and arrangement between mtDNAs (Figures 1A,B,G), and the similarity is also higher (Chen et al., 2011a). Therefore, a small change in mitochondrial genome can make *nap* cytoplasm change into *pol* sterile cytoplasm, resulting in the formation of CMS-inducing gene orf224 in the sterile plant (ZY21A-1, orf224 is the key regulatory gene of pol CMS). There was no orf224 in mitochondria before induction. During the induction, some fragments of mitochondria rearranged, resulting in the emergence of orf224, changing 0933B from fertile to sterile (ZY21A-1). This may explain why *pol* CMS in *Brassica napus* was discovered by Prof. Fu Tingdong in 1973 (Fu, 1981). Moreover, the same results occurred in multiple replicate experiments and were also stably inherited in the F_2_ generation after induction, while indicating that these transposon jumps were targeted and persistent. Although some speculations on the induction mechanism of DH induction lines were made according to current results, the induction mechanism of DH inducer is complex, and whether the real induction mechanism is the same as what we speculated will be explored in more depth.

### Doubled Haploid Induction Lines Provide a New Model for Innovative Germplasm in *Brassica napus*

**FIGURE 6:**
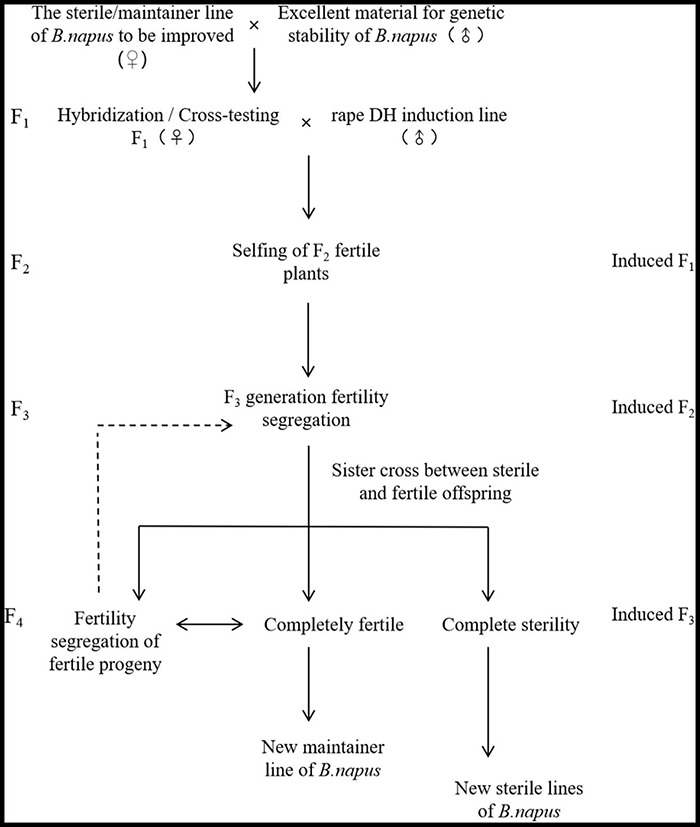
Model diagram of rapid synchronous breeding of maintainer sterile line.

To ensure the reliability of the experiment, the planting process was strictly carried out with sorted harvesting and sorted seedling transplanting and planting. It excluded the possibility that the CMS plants in the induced F_1_ generation were produced by the mixed planting. Meanwhile by repeating the experiment, the CMS plants were also produced during maintainer induction of other genotypes. These results indicate that the phenomenon can be repeatedly realized, ensuring the realization path of the innovative CMS line selection method for DH induction lines. Meanwhile, because of the rapid and simultaneous breeding of maintainers and CMS lines when different genotypes of the female parents are induced by DH induction lines in *B. napus*, a new model for the innovation of germplasm resources is it provided. On the one hand, because the induction effect of the induction lines is influenced by the karyogene and cytoplasm genotype of the female parent, favorable traits are generated and rapid stabilization of traits is carried out by inducing female parents to gain or lose corresponding functions. On the other hand, the induction lines can enhance the SNP purity rate of the plants, and the induction progeny has the characteristics of fertility segregation for rapid and simultaneous breeding for the maintenance and CMS lines. First, the cytoplasmic maintainer or the CMS line of the known *B. napus* to be improved as the female parent and other excellent traits *B. napus* as the male parent to cross or test cross (Figure 6) was used and then DH induction lines were used to pollinate the generated F_1_ generation and induce it to produce karyogene homozygous F_2_ generation (induced F_1_). Subsequently, the excellent F_2_ generation (induced F_1_) to self-crosses were selected and then in the F_3_ generation (induced F_2_), fertile plants without restorer genes and CMS plants with a genetic similarity coefficient greater than 0.90 (genetic distance less than 0.1) to self-crossed and sister-crossed were screened out. Also, the sterility of fully fertile self-crossed plants and sister-crossed sterile lines, were observed when the sister-crossed sterile offspring are completely sterile and the corresponding fully fertile offspring are not separated from the sterile plants, the corresponding maintainer and CMS lines with the consistent nuclear background are formed. Meanwhile, when the offspring of a fully fertile individual plant is still a separated sterile and fertile offspring, the paired sister crosses of multiple individual plants are repeated until no longer separated (Figure 6). This not only enables rapid breeding of new inbred lines but also can obtain homozygous corresponding maintainer and CMS lines in about 2–4 generations at the earliest (Figure 6). This saves a lot of time and manpower compared to the traditional breeding method by saving the process of allelic purification of maintainer lines and backcrossing of maintainer lines with CMS lines for multiple generations and karyogene replacement. Therefore, the application of rape DH induction lines can accelerate and change the breeding pattern of rapeseed and create new ideas for the development of germplasm resources, which have great potential for application and practical value. There are also differences between rape DH induction lines and maize and *Arabidopsis* haploid induced lines in terms of induction function.

## Conclusion

In this study, by examined the parents and offspring before and after induction, we found that when the inducer lines were pollinated as male parents to homozygous females, the nuclear background of the induced F_1_ generation was almost the same as that of the females, and the fertile progeny did not contain fertility restorer genes, but the cytoplasmic type of the sterile progeny changed and was rapidly stabilized in the F_2_ generation. Simultaneous induction of different types of heterozygous females resulted in similar results while indicating that the inducer lines have the ability to obtain both maintainers and sterile lines simultaneously and rapidly. Also, the induction mechanism of inducers may be related to chromosome elimination and nuclear-cytoplasmic interactions, and these findings provide new insights for the innovation of oilseed rape germplasm resources.

## Data Availability Statement

The datasets presented in this study can be found in online repositories. The names of the repository/repositories and accession number(s) can be found in the article/Supplementary Material.

## Author Contributions

SF, WZ, and ZL conceived and designed the experiments. WZ, YZ, and XYL performed the experiments. WZ, HS, and XL carried out data analysis. YL, JY, PX, JW, WG, QZ, LT, ZK, and RT carried out field cultivation. WZ wrote the manuscript. SF and CX edited the manuscript. All authors read and approved the content.

## Conflict of Interest

The authors declare that the research was conducted in the absence of any commercial or financial relationships that could be construed as a potential conflict of interest.

## Publisher’s Note

All claims expressed in this article are solely those of the authors and do not necessarily represent those of their affiliated organizations, or those of the publisher, the editors and the reviewers. Any product that may be evaluated in this article, or claim that may be made by its manufacturer, is not guaranteed or endorsed by the publisher.

## Footnotes

1
https://blast.ncbi.nlm.nih.gov/Blast.cgi

2
http://cbi.hzau.edu.cn/rape/download_ext/zs11.geneinformation.txt

3
https://www.genome.jp/kegg/ko.html
