# Supplementary material for: Rapid and Synchronous Breeding of Cytoplasmic Male Sterile and Maintainer Line Through Mitochondrial DNA Rearrangement Using Doubled Haploid Inducer in Brassica napus
Source: Front Plant Sci. 2022 Apr 26;13:871006. doi: 10.3389/fpls.2022.871006 (PMC9087798; doi:10.3389/fpls.2022.871006)
Supplement: Supplementary file 4 [file Data_Sheet_1.docx]

Supplementary Material

**Title:** Rapid and synchronous breeding of CMS and maintainer line through mitochondrial DNA rearrangement using double haploid inducer in *Brassica napus*

**Supplementary Figure 1.** Identification results of ploidy and chromosome number of hybrid hexaploid progeny ZY26A-1. **(A)** Flow cytometry results of hybrid hexaploid progeny ZY26A-1. **(B)** Number of the chromosome of the heterozygous hexaploid progeny ZY26A-1, Bar=10μm.

**
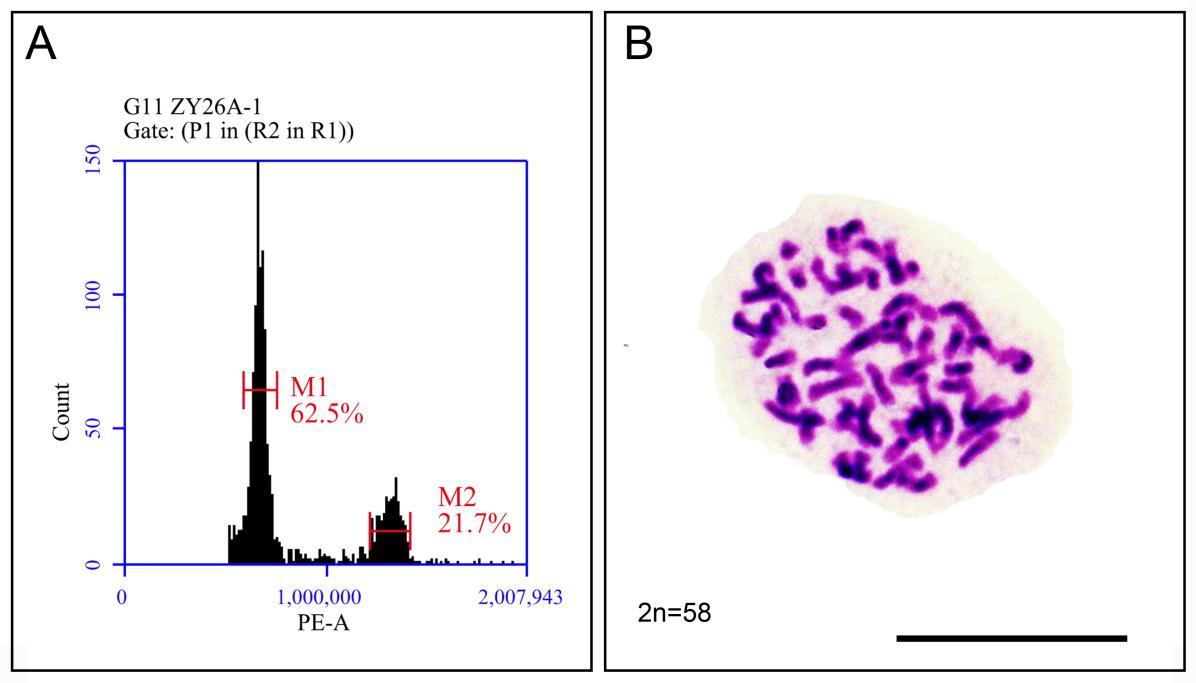
**

**Supplementary Figure 2.** Partial primer sequencing comparison results. **(A)** Comparative results of sequence detection of *pol* CMS recovery gene *Rfp1*. **(B-D)** Sequencing results of mitochondrial genome partial detection primers (mt-1, mt-2, mt-3) on 0933A. The red box represents the location of the primer. The numbers in the sequence represent the number of omitted bases.

**
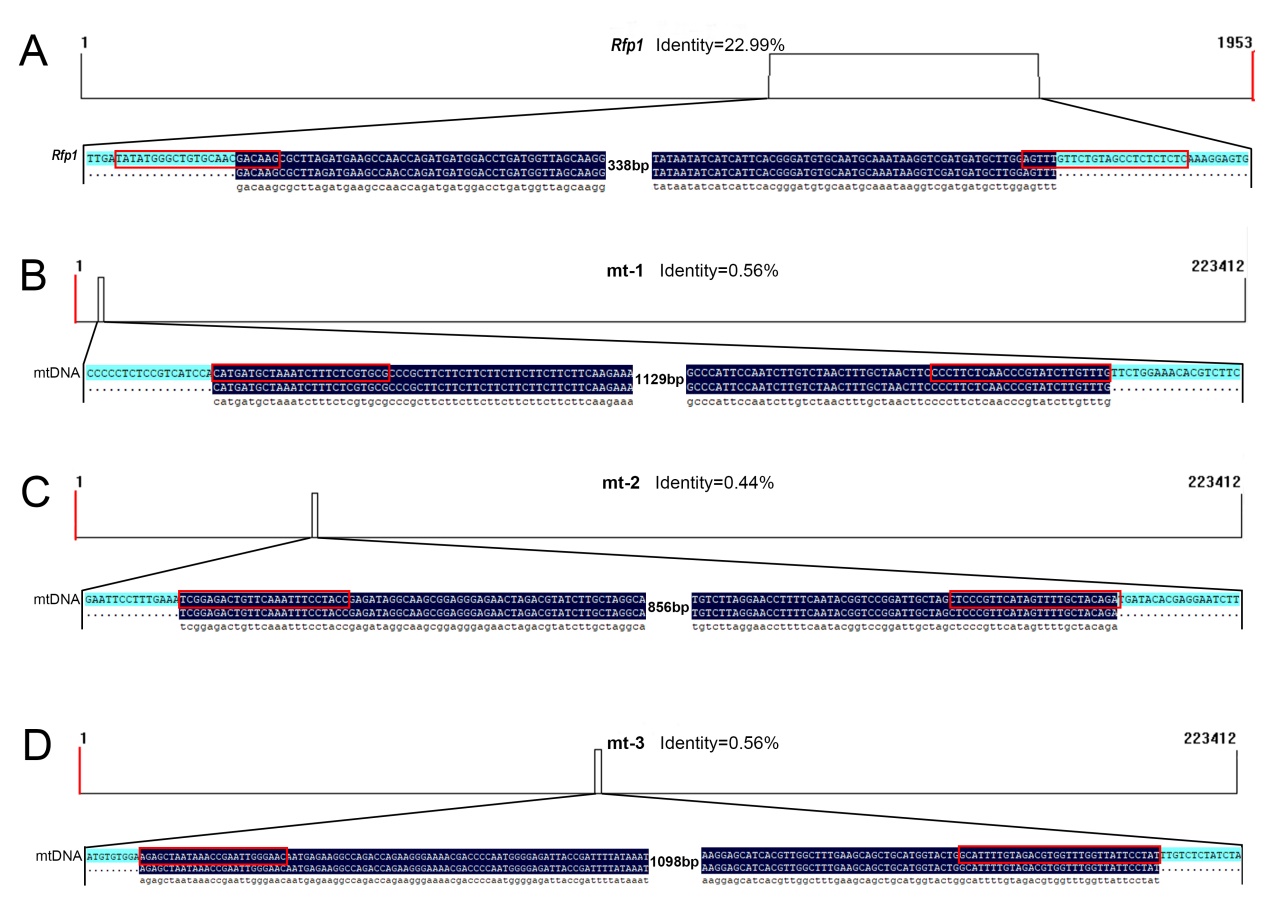
**

**Supplementary Figure 3.** Annotated maps of mitochondrial genomes of some parental and F_1_ generations. **(A-H)** Mitochondrial genome annotation maps for 0933B, 0933A, L0933A, ZY26A-1, Y3380, Y3560, 20-2386, and Z1732, respectively.

**
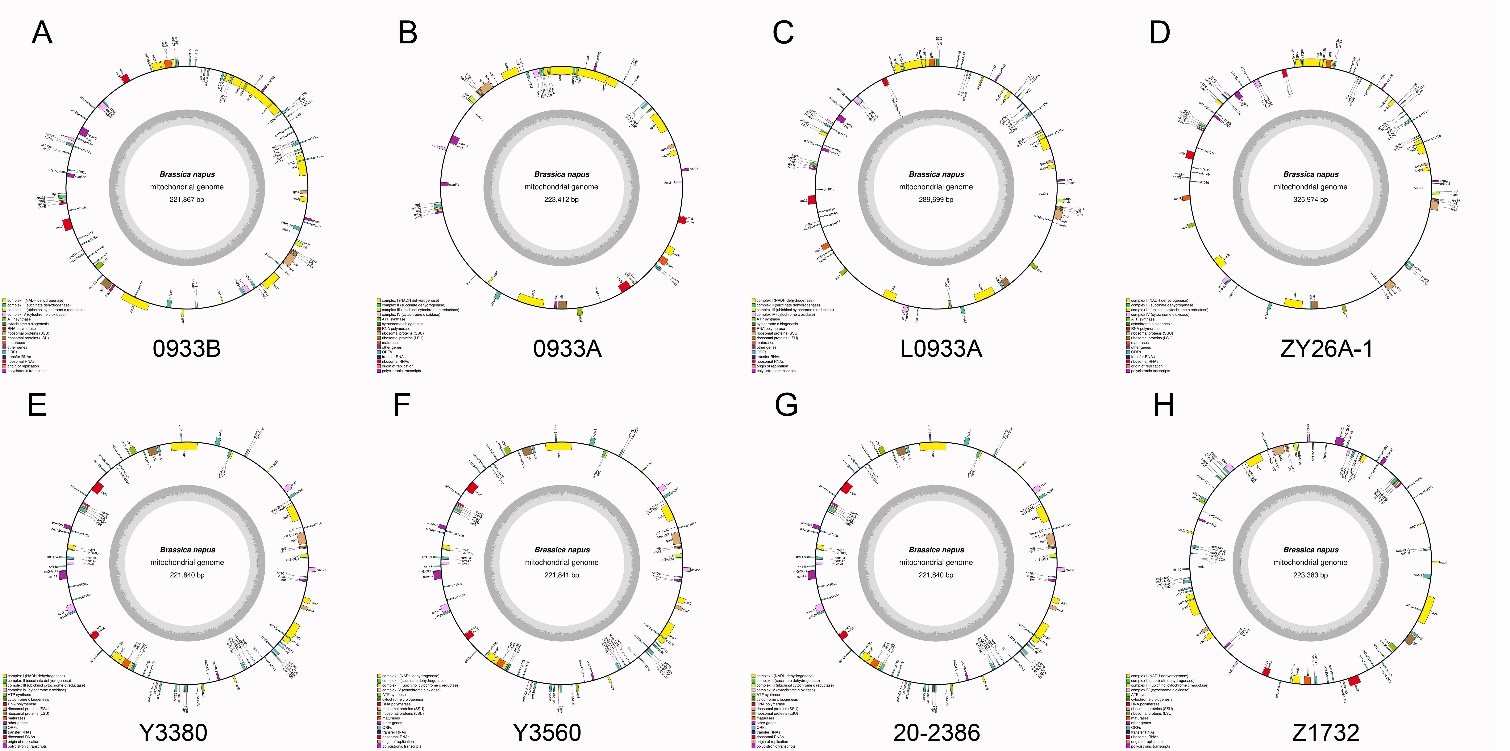
**

**Supplementary Figure 4.** The GO and KEGG Brite enrichment of the gene where the mutant locus is located in the offspring. **(A-C)** The GO enrichment results for ZY21-1, ZY21A-1 and ZY26A-1, respectively. **(D-F)** The KEGG Brite enrichment results for ZY21-1, ZY21A-1 and ZY26A-1, respectively.

**
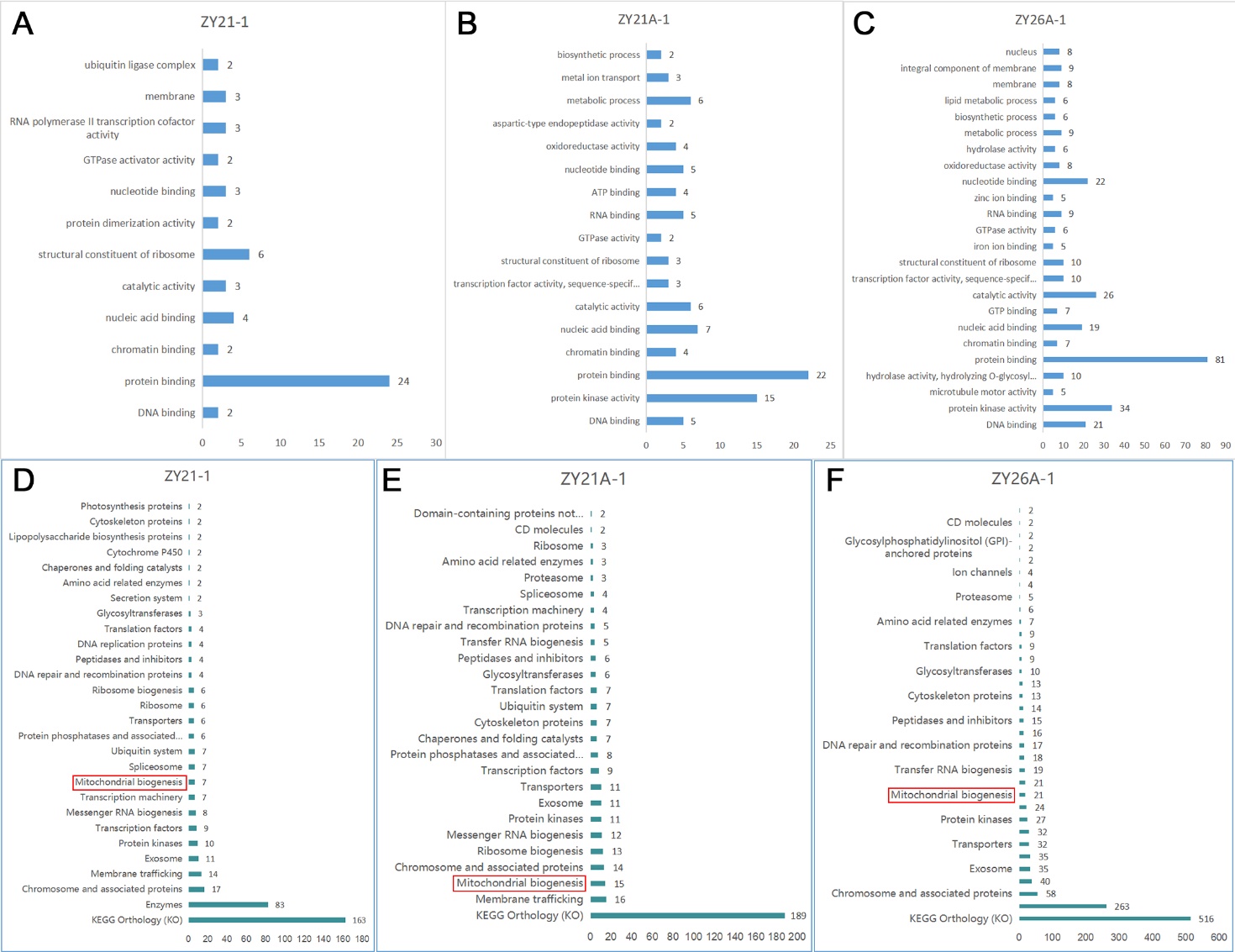
**

**Supplementary Table 1.** Cytoplasmic genotype, restorer gene, mitochondrial genome detection primer.

| Primer name | | Primer sequence (5'-3') | Primer type |
| --- | --- | --- | --- |
| MSS4-F | AGTATTTCGTTCACCTTGGC | Cytoplasmic identification primer |  |
| MSS4-R | GCTTGGTGGAAAAGATCGTA |  |  |
| MSS8-F | GAGAAAGAGAGAAGGTTGCT |  |  |
| MSS8-R | AGCAGAGGAGTGTGCTGACT |  |  |
| MSS14-F | GCTCGTTCGATTAAGCTCAA |  |  |
| MSS14-R | GAATTCCTCTTTCATTGCGG |  |  |
| Actin-F | TGCTCTTCCTCACGCTATCCTC | Internal reference |  |
| Actin-R | GCTCGTAGTTCTTCTCCACCG |  |  |
| OPSNP7-F | TATATGGGCTGTGCAACGACAAG | *Pol* restorer primer |  |
| OPSNP7-R | GAGAGAGAGGCTACAGAACAAACT |  |  |
| mt-F1 | CATGATGCTAAATCTTTCTCGTGCG | Mitochondrial genome sequencing detection primer |  |
| mt-R1 | CAAACAAGATACGGGTTGAGAAGGG |  |  |
| mt-F2 | TCGGAGACTGTTCAAATTTCCTACC |  |  |
| mt-R2 | TCTGTAGCAAAACTATGAACGGGAG |  |  |
| mt-F3 | AGAGCTAATAAACCGAATTGGGAAC |  |  |
| mt-R3 | TAACCAAACCACGTCTACAAAATGC |  |  |

**Supplementary Table 2.** Nei genetic distance of some individual plants in the F_2_ generation population of heterozygous female parent induced by induction lines. The yellow filler was the genetic distance between inducing different heterozygous females produced fertile and sterile individual plants in the same F_2_ generations.
